# Transcriptomic basis for drought-resistance in *Brassica napus* L.

**DOI:** 10.1038/srep40532

**Published:** 2017-01-16

**Authors:** Pei Wang, Cuiling Yang, Hao Chen, Chunpeng Song, Xiao Zhang, Daojie Wang

**Affiliations:** 1 State Key Laboratory of Cotton Biology, School of Mathematics and Statistics, Henan Key Laboratory of Plant Stress Biology, College of Life Sciences, Henan University, Kaifeng, Henan, China

## Abstract

Based on transcriptomic data from four experimental settings with drought-resistant and drought-sensitive cultivars under drought and well-watered conditions, statistical analysis revealed three categories encompassing 169 highly differentially expressed genes (DEGs) in response to drought in *Brassica napus* L., including 37 drought-resistant cultivar-related genes, 35 drought-sensitive cultivar-related genes and 97 cultivar non-specific ones. We provide evidence that the identified DEGs were fairly uniformly distributed on different chromosomes and their expression patterns are variety specific. Except commonly enriched in response to various stimuli or stresses, different categories of DEGs show specific enrichment in certain biological processes or pathways, which indicated the possibility of functional differences among the three categories. Network analysis revealed relationships among the 169 DEGs, annotated biological processes and pathways. The 169 DEGs can be classified into different functional categories via preferred pathways or biological processes. Some pathways might simultaneously involve a large number of shared DEGs, and these pathways are likely to cross-talk and have overlapping biological functions. Several members of the identified DEGs fit to drought stress signal transduction pathway in *Arabidopsis thaliana*. Finally, quantitative real-time PCR validations confirmed the reproducibility of the RNA-seq data. These investigations are profitable for the improvement of crop varieties through transgenic engineering.

*Brassica napus L*., (*B. napus*, also known as oilseed rape), is an allopolyploid plant formed by hybridization between *B. rapa* and *B. oleracea*, and subsequent chromosome doubling^1^. The genome of *B. napus* consists of *A*_*m*_*A*_*m*_*C*_*n*_*C*_*n*_. The genome of *B. rapa* consists of *A*_*r*_*A*_*r*_, whereas *B. oleracea* consists of *C*_*o*_*C*_*o*_. *B. napus* is cultivated mainly for its oil-rich seed. Oilseed rape is an economically important crop. According to a report from the United States Department of Agriculture, *B. napus* is the third-largest source of vegetable oil in the world. Drought is an abiotic stress factor that severely affects plant growth and agricultural production^2^,^3^,^4^,^5^,^6^,^7^,^8^,^9^,^10^,^11^,^12^. The breeding of disease- and drought-resistant varieties are long-term goals, many of which have been met using genetic engineering^7^. In 2009, it was reported that 90% of the rapeseed crops planted in Canada were genetically modified, herbicide-tolerant varieties^13^. Therefore, the identification of drought-resistant genes and the evaluation of their potential functions in stress adaptation are an important step in further improving crop stress tolerance.

With the rapid development of high-throughput technologies, many researchers have investigated stress-mediated differences in the gene expression of some organisms by using microarrays^14^,^15^,^16^,^17^,^18^,^19^,^20^,^21^,^22^,^23^. Microarray datasets include up to tens of thousands of genes with relatively small sample numbers; however, not all of the genes are related to the purpose of the study. Biologically, there are often tens to hundreds of genes that are significantly associated with traits such as drought and salt resistance. In plants, several drought-resistant mechanisms are associated with leaf traits, and these traits are often used as criteria to evaluate the drought resistance of crops. Morphological traits, such as leaf rolling, stomatal density, and stomatal aperture control, along with physiological traits such as cuticular wax content, abscisic acid (ABA) content, relative water content, water potential, and canopy temperature, are frequently used as criteria for drought avoidance. Osmotic adjustment, cell membrane stability, proline and sugar content, among others, are generally considered to be criteria for drought tolerance. Drought avoidance and drought tolerance are two major mechanisms underlying drought resistance^7^,^24^; the two concepts have frequently been interpreted as being equivalent^7^. To date, some mechanisms have been proposed to explain the drought response of plants. To respond to drought stress, plants use multiple strategies and have evolved to adapt to drought^7^ by, for example, shortening their life cycle with accelerated flowering, reducing water loss via stomatal closure and increasing the thickness of the leaf cuticle, improving water uptake by developing a deep and thick root system, and accumulating osmoprotectants, antioxidants, and reactive oxygen species (ROS) scavengers. Plant responses to drought stress include stress signal perception, signal transduction and amplification, and adaptation at the morphological, physiological, and molecular levels^7^, which are complex processes.

Here, we first provide a brief overview of several investigations of drought resistance in plants such as rice, *Arabidopsis thaliana* and maize. Next, we review some recent studies on *B. napus*. In 2009, Degenkolbe *et al*.^15^ investigated the gene expression profiles of two drought-tolerant rice varieties and two drought-sensitive rice varieties under drought stress conditions. They found that only 245 and 413 genes were consistently down-regulated and up-regulated, respectively, among all four cultivars. In 2011, by applying the feature elimination method based on the support vector machine, Liang *et al*.^21^ predicted drought-resistant genes in *Arabidopsis thaliana*. On the basis of 22 sets of gene expression data for *Arabidopsis thaliana* from the GEO database (http://www.ncbi.nlm.nih.gov/geo/), the authors predicted key genes involved in drought tolerance. They detected the top 10 predicted genes and found that seven genes were relevant to biological processes during drought resistance. In 2012, Kakumanu *et al*.^22^ investigated the effect of drought on gene expression in maize reproductive and leaf meristem tissue by using RNA-seq. They found that more drought-responsive genes responded in the ovary compared with the leaf meristem. Regarding *B. napus*, because its complete genomic sequence was not published until 2014 by Chalhoub *et al*.^1^, investigations of *B. napus* at the -omics level remain scarce^7^,^8^,^20^,^25^,^26^,^27^,^28^,^29^. In 2014, Yong *et al*. performed comparative transcriptome analysis of leaves and roots of *B. napus* in response to a sudden increase in salinity by RNA-Seq. A total of 582 transcription factors and 438 transporter genes were differentially regulated in both organs in response to salt shock. Several important pathways involved in the salt-stress signal transduction have been identified^26^,^30^,^31^,^32^. Recently, Zhang *et al*.^33^ have identified 16 loci that are significantly associated with the water stress response, by using a genome-wide association study (GWAS). By combining the DEGs detected by RNA-seq with significantly associated loci from the GWAS, the authors have identified 79 candidate genes that play a role in water stress tolerance in *B. napus*, among which eight are putatively associated with drought tolerance on the basis of the gene ontology (GO) of *Arabidopsis thaliana*. The associated studies provide some insights into the stress response of *B. napus*.

Although great advances have been achieved over the past few decades, and some drought-resistant varieties have been bred^7^, the general molecular basis of drought-resistance is still not well understood^7^,^8^,^34^, especially for *B. napus*. For molecular breeding of new drought-resistant crops and transgenic engineering, it is interesting and important to design appropriate experiments to identify drought-resistant genes in *B. napus*. However, to our knowledge, only a few studies have been conducted to explore drought-responsive genes in *B. napus*^8^,^33^,^35^. Motivated by these deficiencies, the aim of this paper was to explore the identification and analysis of drought-resistant genes in *B. napus*.

## Results

### Transcriptome sequencing results

We considered four samples for transcriptome sequencing: S, ST, R and RT. ST and RT are two *B. napus* varieties that exhibit opposite physiological phenotypes after PEG-6000 treatment^35^,^36^,^37^,^38^. S and R correspond to samples ST and RT treated with 200 g *L*^−1^ PEG-6000. Statistical analysis of the transcriptome sequencing results for the four samples are shown in Table 1. More than 6.6 × 10^4^ genes were expressed in each of the four samples, among which the clean reads and genome map rates were all higher than 77% and 60%, respectively. Detailed descriptions of the transcriptome sequencing data can be found in our previous work^35^.

### Variety-specific gene expression within comparison groups

**Figure 1:**
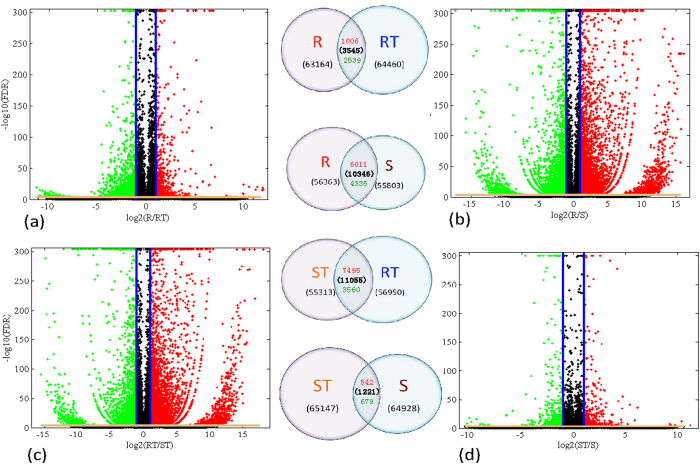
Volcano plots for expressed genes in the four comparison groups. Volcano plots for all the expressed genes in (**a**) RT-R, (**b**) S-R, (**c**) ST-RT, and (**d**) ST-S. X- and Y-axis present the *log*_2_(*ratio*) for the two samples and −*log*10(*FDR*), respectively. Red (Up regulated) and green (down regulated) dots mean that the genes have significant difference (*FDR* ≤ 0.001, and at least with 2 fold differences, namely, |*log*_2_(*ratio*)| ≥ 1), while the dark dots correspond to genes with no significant differences. The two vertical blue lines in each panel correspond to *log*_2_(*ratio*) = 1 and *log*_2_(*ratio*) = −1, respectively. The yellow horizontal line represents *FDR* = 0.001, namely, −*log*10(*FDR*) = 3. The inner Venn diagrams shows the numbers of expressed genes for the four comparison groups. Number of the DEGs in each group are listed in parentheses, numbers above and below the parentheses denote up- and down-regulated genes, respectively.

Using the four samples, we constructed four comparison groups: ST-S, S-R, ST-RT and ST-S. Figure 1 shows a scatter plot of the *log*2-fold change versus the −*log*10(*FDR*) for the four groups, and the corresponding Venn diagrams^39^ for the four comparison groups are also shown. By restricting *FDR* ≤ 0.001 and |*log*_2_(*Ratio*)| ≥ 1, the four comparison groups contained 3545, 10346, 11055, and 1221 DEGs, respectively. This finding indicates that there are fundamental differences in gene expression levels within varieties and under stress conditions. A comparison between RT-R and ST-S revealed 1006 up-regulated and 2539 down-regulated DEGs in RT-R, compared with 542 up-regulated and 679 down-regulated DEGs in ST-S. In RT-R and ST-S, more DEGs were down-regulated after drought exposure. Furthermore, under drought conditions and compared with the drought-sensitive cultivar, many more genes in the drought-resistant cultivar were stress responsive.

In the comparison groups with two different cultivars, 6011 up-regulated and 4335 down-regulated DEGs were detected in S-R compared with 7495 and 3560 in ST-RT, respectively. On the one hand, this finding indicates that the DEGs in the comparison groups with two different cultivars (Fig. 1(b,c)) greatly exceed those in groups with the same cultivar (Fig. 1(a,d)), and transcriptome differences were observed between the two cultivars. On the other hand, the S-R and ST-RT comparison groups all had many up-regulated DEGs, thus indicating that the gene expression patterns in the two comparison groups varied both under drought stress and normal water conditions. More DEGs tended to be up-regulated in the drought resistance cultivar than in the sensitive one.

### Identification and classification of DEGs

**Figure 2:**
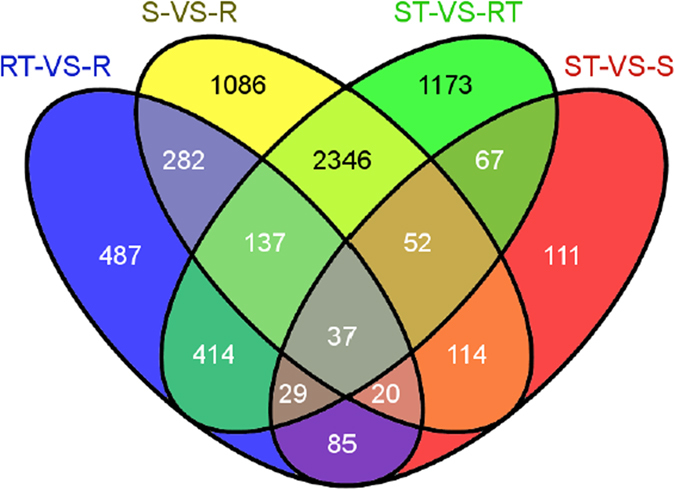
Venn diagrams for DEGs with pathway annotations in the four comparison groups.

Owing to the limited gene annotation for *B. napus*, many DEGs currently lack known function or pathway annotations. The genes with pathway annotations were used for further analysis. After DEGs without pathway annotations were filtered out, only 1491, 4074, 4255 and 515 DEGs remained in the ST-S, S-R, ST-RT and ST-S groups, respectively, (Supplementary Information Table S1). The Venn diagram for the DEGs remaining in the four groups is shown in Fig. 2. The four groups consisted of 15 disjointed subgroups. In all four groups, there was a total of 6440 unique DEGs, among which there were 7.56% (487/6440), 16.86% (1086/6440), 18.21% (1173/6440), and 1.72% (111/6440) group-specific DEGs. Only 37 DEGs were commonly expressed across all four groups.

Among the 15 sets of DEGs, because ST and RT were not all treated by drought stress, the comparison group ST-RT was not included in the analysis. After exclusion of the eight subgroups contained in ST-RT, only seven subgroups remained. The information for the seven DEG subgroups is shown in Table 2. The seven subgroups contained a total of 2185 unique drought-responsive DEGs. The seven subgroups were further classified into three categories: genes from the resistant variety with drought-responsive (RDR), genes from the sensitive variety with drought-responsive (SDR), and common (non-specific varieties) drought-responsive (CDR) DEGs. These three categories consisted of 769, 225 and 1191 DEGs, making up 11.94%, 3.49% and 18.49% of the 6440 DEGs, respectively.

**Figure 3:**
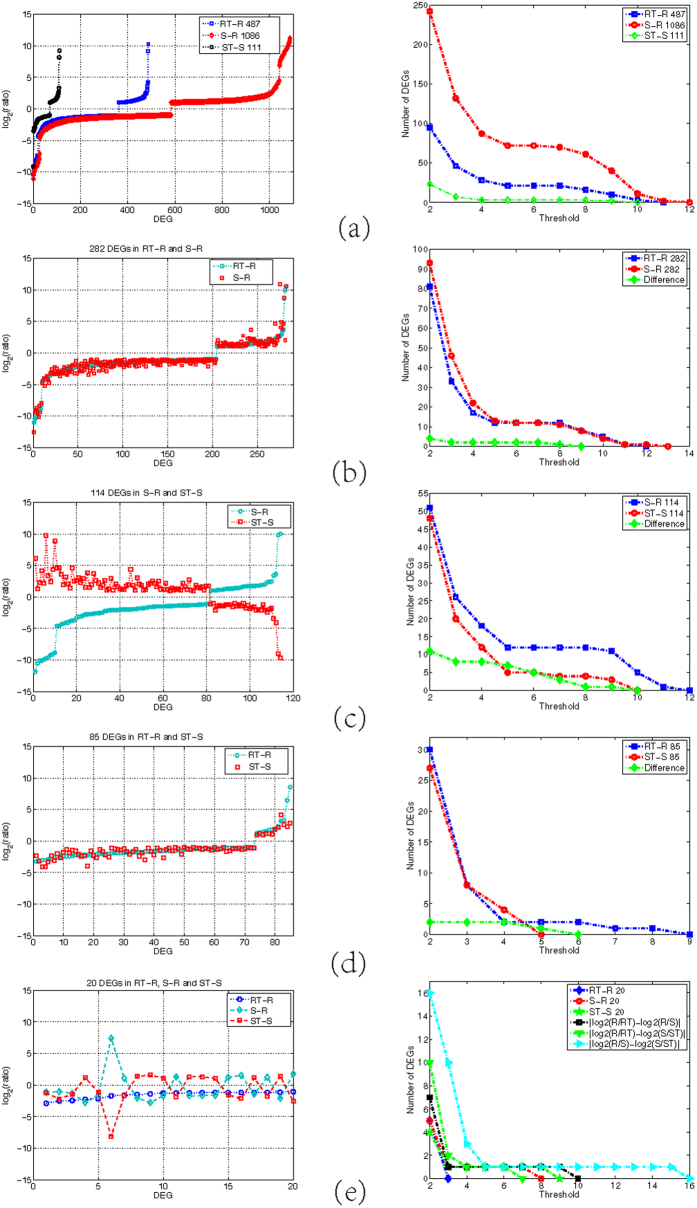
*Log*_2_ ratio and absolute *log*_2_ ratio profiles of the seven subgroups of susceptible drought-responsive DEGs. The left panels show the *log*_2_ ratio value for each gene. The right panels show the corresponding absolute *log*_2_ ratio distributions as well as the curves of the differences among groups. (**a**) The three subgroups of unique DEGs in RT-R, S-R and ST-S. (**b**–**d**) The three subgroups of DEGs consist of the intersections of two comparison groups. (**e**) The 20 common DEGs in RT-R, S-R and ST-S.

Subsequently, we mainly explored the three categories of DEGs and identified crucial drought-responsive genes unless otherwise noted. First, we investigated the expression profiles of the absolute *log*_2_ fold change for the three classes of DEGs. Because some DEGs were involved in more than one comparison group, we displayed their *log*_2_(*ratio*) profiles in each comparison group in the left panels of Fig. 3. The right panels in Fig. 3 show the absolute *log*_2_ fold change distributions, as well as the absolute differences among groups if the considered DEGs appeared in more than one comparison groups. Figure 3(a) shows that by excluding the 85 DEGs in RT-R and ST-S and the 20 DEGs in RT-R, S-R and ST-S, the other five DEG subgroups all exhibited wide distributions in *log*_2_ fold change values, with differences greater than 10-fold in some groups.

Second, on the basis of Fig. 3, we performed a second round of DEG selection. Considering both the *log*_2_ fold change values for each DEG and the absolute differences among different comparison groups, we selected the DEGs to identify most crucial ones. Detailed conditions for the filtering are shown in Table 3, where we also present information for the final selection of 169 susceptible crucial DEGs (for details, also see Supplementary Information Table S2). Of the 169 DEGs, 37 were RDR, 35 were SDR and 97 were CDR, with CDR making up more than one half.

### Locations of DEGs show no chromosome preference

**Figure 4:**
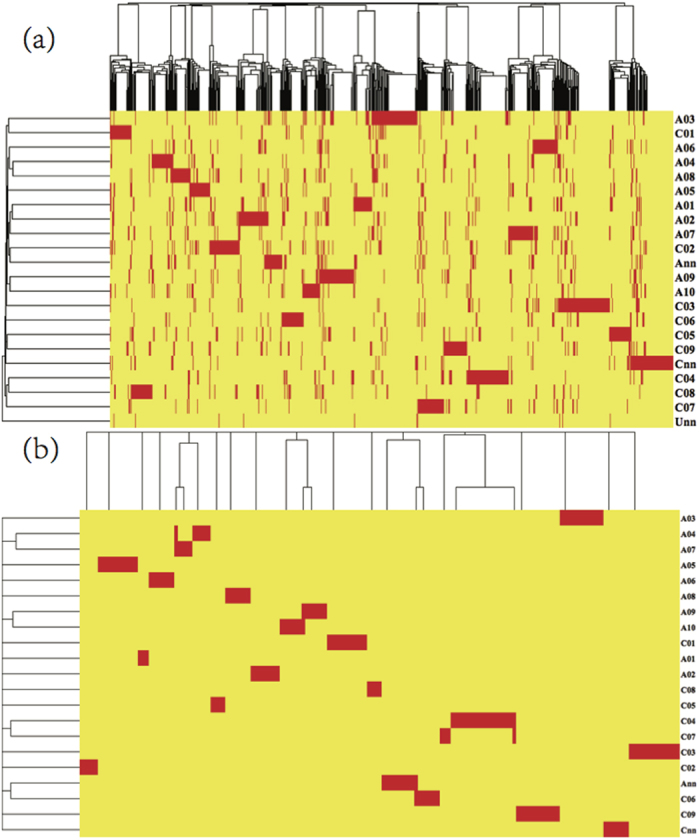
Clustering dendrograms for the locations of the 6440 DEGs and the 169 susceptible crucial drought responsive DEGs on its 19 pairs of chromosomes. (**a**) Case for the 6440 DEGs. (**b**) Case for the 169 crucial DEGs. For completeness, we also considered the DEGs without known chromosome locations, that is, with chromosome labels Ann, Cnn and Unn. The Pearson correlation coefficients and the average linkage method^71^ are used during the hierarchical clustering processes.

*B. napus* has 19 pairs of chromosomes, denoted A01-A10, C01-C09. To determine whether the DEGs were preferentially located on specific chromosomes, we constructed clustering dendrograms^40^,^41^ of the locations of the 6440 DEGs and the 169 susceptible crucial DEGs on the chromosomes, as shown in Fig. 4.

The DEGs were located at chromosome positions from 20 to 78710. The clustering trees for the columns in Fig. 4 indicated that the DEGs tended to be cluster at certain positions in different chromosomes. On the basis of the clustering dendrogram shown in panel (a) of Fig. 4, the 6440 DEGs were fairly uniformly distributed on different chromosomes, similarly to the 169 crucial DEGs (Panel (b) of Fig. 4), thus indicating that the selected DEGs were also uniformly distributed throughout the chromosomes. These results indicate that it is generally difficult to breed new drought-resistant cultivars via genetic engineering at a single or a few genic locations. Thus, the mining of drought-responsive DEGs will be a difficult task, and not only a few drought-responsive DEGs should be identified.

### Differential expression patterns of the selected DEGs

**Figure 5:**
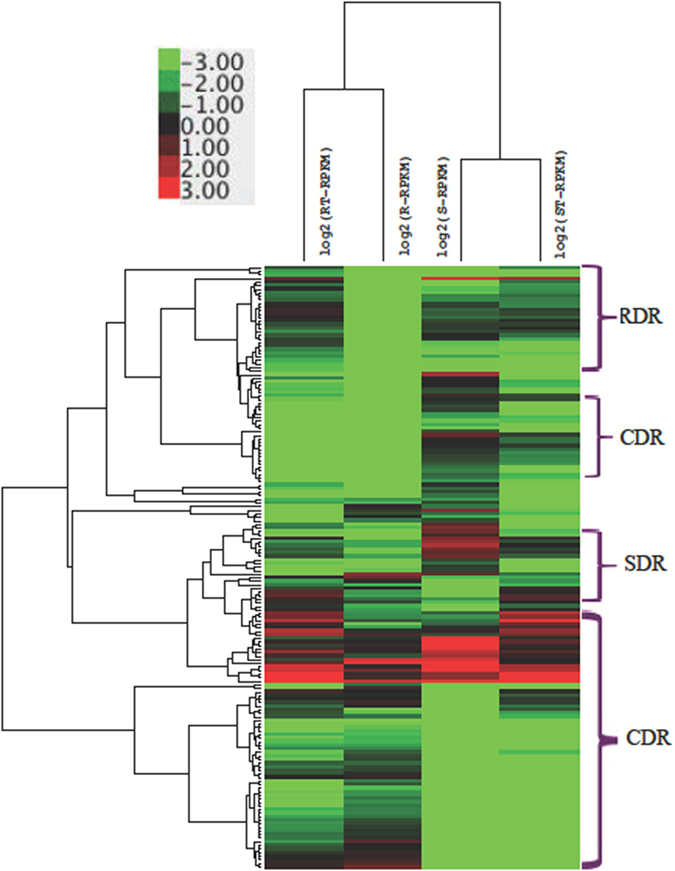
Clustering of the expression patterns for the 169 DEGs in R, RT, S and ST. The clustering analysis is based on the *log*_2_(*RPKM*) values of each gene in the four samples. The Euclidean distance and the average linkage method are used in the hierarchical clustering analysis.

To explore the expression patterns of the 169 DEGs on the basis of the log RPKM values^42^ in the four samples (R, RT, S and ST), the clustering dendrogram is shown in Fig. 5. As shown in Fig. 5, on the one hand, the expression levels of the three groups of DEGs (RDR, SDR and CDR) exhibited some clustering features. On the other hand, the expression levels of the 169 DEGs for the same cultivar were similar. These results indicate that species from the same cultivar tend to have similar expression, even during exposure to different treatments.

In fact, for the RDR shown in Table 2, 74.33% of the 487 unique DEGs in RT-R were down-regulated. Among the 282 common DEGs in RT-R and S-R, 204 (72.34%) were down-regulated. Among the selected 37 RDR, 29 (78.38%) genes were down-regulated in R compared with RT. The results indicate that the expression of the selected RDR in R tends to be down-regulated after exposure to drought stress. Regarding the 225 SDR obtained after the first round of filtering, 70 of the 111 unique ST-S genes were down-regulated; among the 114 common DEGs in ST-S and S-R, 81 were up-regulated in ST-S but down-regulated in S-R. In the second, 35 crucial SDR were selected, among which 30 were up-regulated in S compared with ST, whereas 28 genes were up-regulated in S compared with R. This result indicates that the selected SDR tends to be down-regulated in S compared with ST, whereas the selected SDR tends to be up-regulated in S compared with R. Regarding the 1191 CDR after the first round of filtering, 583 of the 1086 genes that were unique to R were down-regulated compared with S, 73 of the 85 DEGs in RT-R and ST-S were down-regulated, and most of the 20 DEGs common to RT-R, S-R and ST-S were also down-regulated, thus indicating that the expression of CDR tends to be down-regulated after drought exposure.

### GO enrichment analysis

We performed GO enrichment analysis and, for simplicity, considered mainly GO biological processes^43^. For all screened DEGs in the four comparison groups, biological process enrichment analysis revealed that all four groups were commonly enriched in various responsive processes and defense responses, such as the response to oxygen-containing compounds, cold, lipids, nitrate, nitrogen compounds, nutrient levels, organic substances, osmotic stress, salt stress, other organisms, oxidative stress, starvation, water deprivation, wounding, and abiotic, biotic, chemical, endogenous, external, extracellular, hormone, and water stimuli. Interestingly, we found that some biological processes were specifically enriched in certain groups. For example, the term “response to salicylic acid stimulus” was specifically enriched in S-R and ST-S. The term “response to ABA stimulus” was enriched in ST-S, S-R, and RT-R, but it was reduced in ST-RT. Interestingly, many of the identified genes involved in these pathways were down-regulated in sample R compared with RT or S, and many of the commonly enriched processes or specifically enriched processes were related to drought stress^35^.

For the selected 37 DEGs in RDR, 34 were involved in 521 different biological processes, among which 21 DEGs and 16 DEGs were involved in responses to stimuli and stress, respectively. Of the 21 DEGs involve in the response to stimuli, 18 were down-regulated in sample R compared with RT. Fourteen of the 16 DEGs that were responsive to stress were down-regulated in sample R compared with RT. Moreover, more than 5 genes were involved in the response to chemical, abiotic, and hormone stimuli, oxidative stress and many other metabolic processes. Two DEGs and one DEG were involved in the response to osmotic stress and to the ABA stimulus, respectively. Most of the stimulus- and stress-responsive DEGs were down-regulated in sample R compared with sample RT.

Among the 35 DEGs in SDR, 33 had biological process annotations. Among 33 DEGs, 30 and 28 genes were involved in metabolic processes (30/33) and stimulus responses (28/33), and 33 DEGs in SDR were also enriched in cellular processes (27/33) and responses to various other stimuli, such as chemical (21/33), stress (20/33), abiotic (16/33) and hormone (10/33) stimuli. Four DEGs in SDR in particular were involved in responses to osmotic stress and salt stress, respectively, 3 DEGs were involved in the response to oxidative stress, and 3 DEGs were involved in the response to the ABA stimulus. Among the 28 DEGs that were responsive to a stimulus, 23 were down-regulated in sample R compared with RT, but 24 DEGs were up-regulated in sample S compared with ST. Similarly, most of the DEGs involved in the response to stress were also down-regulated in sample R compared with RT but up-regulated in sample S compared with ST.

Among the selected 97 DEGs in CDR, 77 and 72 of the 88 annotated DEGs were involved in metabolic and cellular processes, respectively. Additionally, more than 60% of the DEGs participated in organic metabolic, cellular metabolic, primary metabolic, single-organism and stimulus response processes (53/88). The selected DEGs were also enriched in responses to stress and chemical, abiotic (23), and hormone stimuli (13). Notably, 8, 6 and 5 of the 97 DEGs were related to the response to osmotic stress, oxidative stress and ABA stimuli, respectively. Among the 36 DEGs involved in the response to stress, 13 and 18 DEGs were up-regulated and down-regulated in R compared with S, respectively, thus supporting a crucial role for CDR in the stress response with no significant preference for up-regulation or down-regulation after exposure to drought stress.

In summary, the crucial DEGs showed some enrichment in certain GO biological processes. These DEGs were especially enriched in the response to various stimuli and stress. The responses to stimuli and stress illustrate that the selected DEGs were actually functionally relevant to drought stress^35^. Interestingly, we found that the DEGs in RDR and SDR that were responsive to stimuli and stress tended to be down-regulated in sample R compared with RT, whereas they tend to be up-regulated in sample S compared with ST. However, in CDR, there were no differences in the numbers of down-regulated and up-regulated DEGs in response to stress. The associated GO analysis also indicated the possibility of functional differences among the three categories of crucial drought-responsive DEGs.

### KEGG pathway enrichment and involvement analysis

**Figure 6:**
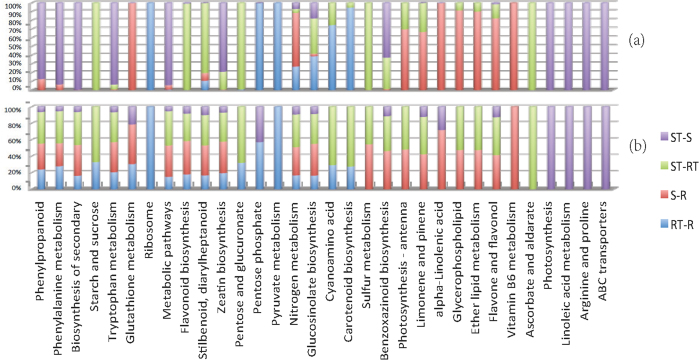
Comparison of the 32 enriched pathways for all the DEGs from the four comparison groups. (**a**) Sectional bar-diagram of FDR values for the 32 enriched pathways. For each pathway, the length of each section of a bar equals to the ratio of FDR in a group to the sum FDR values in the four groups. (**b**) Sectional bar-diagram of frequency of DEGs for the 32 pathways. Higher frequency indicates more preference of the pathways for DEGs. For each pathway, the lengths of the four sections of a bar reflect the relative frequency of DEGs involved in the pathway in the four comparison groups. Longer bar indicates relatively more DEGs in the corresponding group.

The 6440 unique DEGs in the four comparison groups were involved in 128 unique KEGG pathways^44^. Thirty-two pathways were enriched in at least one of the comparison group (*FDR* < 0.05). Comparisons among the four groups in the 32 pathways are shown in Fig. 6, and Fig. 6(a) shows a comparison of the FDR values (the smaller, the better) and (b) shows a comparison of the fractions of related DEGs. Some pathways were enriched in all four groups, such as the biosynthesis of secondary metabolites, flavonoid biosynthesis, glucosinolate biosynthesis, metabolic pathways, nitrogen metabolism, phenylalanine metabolism, phenylpropanoid biosynthesis, stilbenoid, diarylheptanoid and gingerol biosynthesis, tryptophan metabolism, and zeatin biosynthesis. In contrast, some other pathways were enriched in only certain groups. For example, ribosome and pyruvate metabolism were enriched in only RT-R. Vitamin B6 metabolism was enriched in only S-R. Ascorbate and aldarate metabolism were enriched in only ST-RT. Photosynthesis, linoleic acid metabolism, arginine and proline metabolism, and ABC transporters were enriched in only ST-S. For details of the results of the pathway enrichment analysis, one can refer to Supplementary Information Table S3.

The 37 DEGs in RDR involved 31 different pathways, among which more than 3 DEGs were involved in metabolic pathways, the biosynthesis of secondary metabolites, plant-pathogen interactions, phenylpropanoid biosynthesis, starch and sucrose metabolism, phenylalanine metabolism, and plant hormone signal transduction, among others. The 35 DEGs in SDR were involved in 36 different pathways, among which more than 5 DEGs were involved in metabolic pathways, the biosynthesis of secondary metabolites, and alpha-linolenic acid metabolism. The 97 DEGs in CDR participate in 75 different pathways, with 41.23% genes involved in the metabolic pathways. Other identified pathways were the biosynthesis of secondary metabolites, plant-pathogen interactions, phenylpropanoid biosynthesis, plant hormone signal transduction, starch and sucrose metabolism and glutathione metabolism, each of which was associated with at least 5 crucial DEGs.

**Figure 7:**
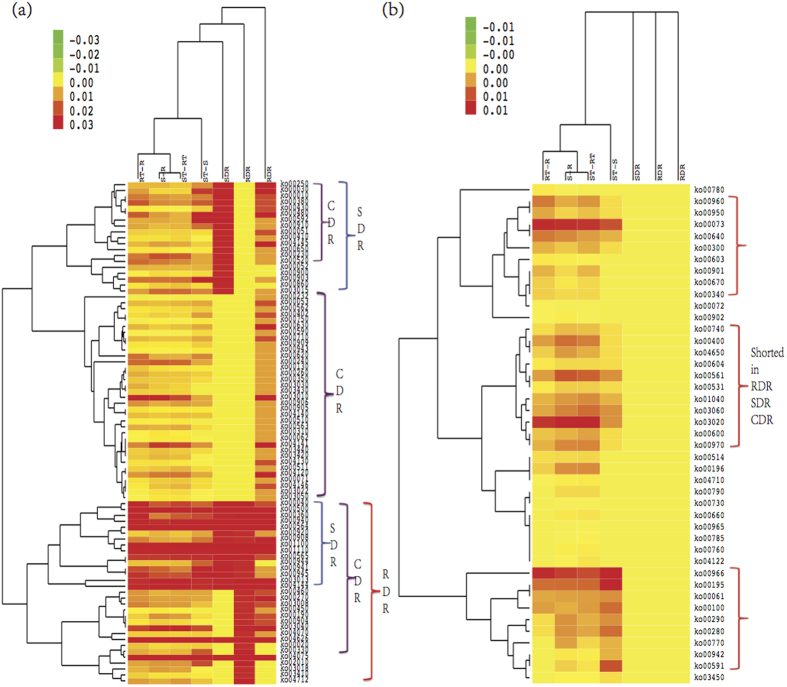
Pathway enrichment analysis based on the clustering analysis. (**a**) The clustering dendrogram for the 85 different pathways that the 169 crucial DEGs involve in. (**b**) The clustering dendrogram for the other 43 different pathways that the 169 crucial DEGs do not involve in. As a comparison, we also show the corresponding results for the 3545, 10346, 11055, 1221 DEGs in the four comparison groups. All the 6440 different DEGs involve in totally 128 unique pathways. The data for the clustering analysis is based on the ratio of genes that related to the considered pathways. During the clustering processes, the distances between different groups and between different pathways are based on the Spearman rank correlation, and the clustering algorithm is based on the average linkage method^71^.

The 169 crucial drought-responsive DEGs were involved in 85 unique pathways. To intuitively explore the pathway involvement of the 169 crucial DEGs, we drew a clustering dendrogram for the 85 pathways encompassing the 169 DEGs as well as a clustering dendrogram for the other 43 pathways that were not involved with the 169 DEGs, as shown in Fig. 7. As shown in Fig. 7(a), we observed that S-R and ST-RT were most similar, whereas the pathways involved in the three classes of crucial DEGs were all very different from the four comparison groups, and the greatest difference was observed for CDR. The 85 pathways were classified into several groups, among which some groups were enriched with certain categories of DEGs, as shown in Fig. 7. In particular, the results showed that ko00020: citrate cycle, ko04712: circadian rhythm-plant, ko00330: arginine and proline metabolism, ko02010: ABC transporters, ko03410: base excision repair, and ko03018: RNA degradation were present specifically in RDR. The pathways ko00052: galactose metabolism, ko00860: porphyrin and chlorophyll metabolism, and ko00900: terpenoid backbone biosynthesis showed specific involvement in SDR. The CDR showed specific involvement in ko04146: peroxisome, ko04141: protein processing in the endoplasmic reticulum and 33 other pathways. All three categories of crucial DEGs are commonly involved in ko01100: metabolic pathways, ko01110: biosynthesis of secondary metabolites, ko04075: plant hormone signal transduction, ko00941: flavonoid biosynthesis, ko04626: plant-pathogen interactions and 11 other pathways. Many of the mentioned pathways have been reported to participate in cross-talk with drought-responsive pathways in plants^15^,^21^,^22^,^35^, such as ko01110: biosynthesis of secondary metabolites, ko04075: plant hormone signal transduction, ko00941: flavonoid biosynthesis, ko02010: ABC transporters, and ko04146: peroxisome.

The three sets of crucial DEGs all showed a reduced involvement in RNA polymerase, cutin, suberine and wax biosynthesis, photosynthesis and glucosinolate biosynthesis. In fact, the reduced pathways were not crucially associated with the drought response, but they were all relevant *in vivo* mechanisms. The KEGG pathway analysis indicated that the associated pathways might have some functional roles during the response to drought stress.

### Network analysis of the 169 DEGs, its biological processes and pathways

**Figure 8:**
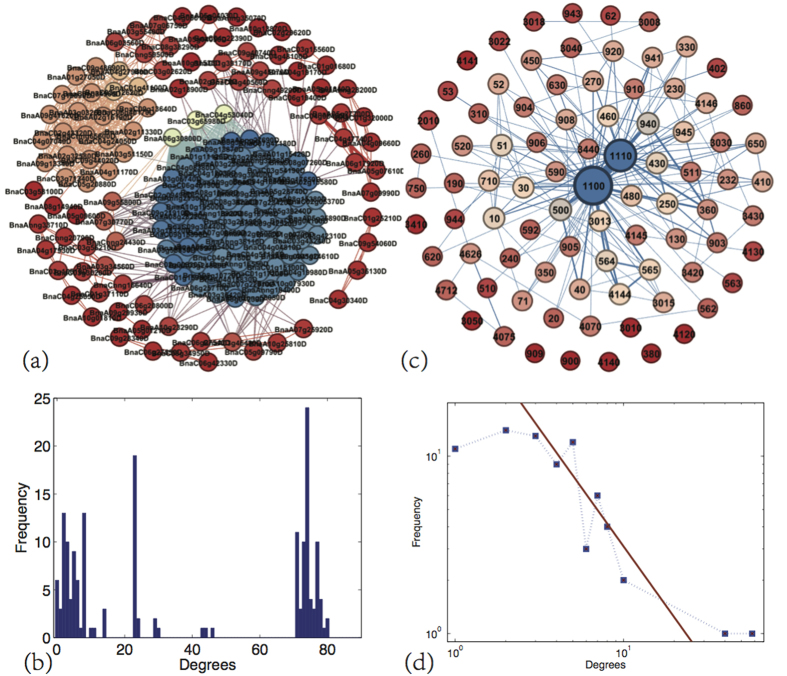
The pathway-gene network and the gene-pathway network for the 169 crucial DEGs. (**a**) The pathway-gene network. Nodes correspond to the 169 genes, if two genes share one or more pathways, we endow a connection between them. Nodes with different colors are with different degrees. (**b**) Degree distribution for the pathway-gene network in (**a**). (**c**) The gene-pathway network for the 85 unique pathways that the 169 DEGs involved. Nodes represent the 85 pathways, if two pathways involved in the same gene, then connect the two pathways by an edge. Numbers in panel (c) represent the pathway numbers. The thickness of an edge between two node in the two networks is proportion to the number of common DEGs involve in the two pathways. (**d**) The corresponding degree distributions of the gene-pathway network as shown in (**c**).

Using the method described in the Materials and Methods section, we constructed several networks for the 169 DEGs. The constructed pathway-gene network and the gene-pathway network for the 169 crucial DEGs are shown in Fig. 8. Figure 8(a) shows that the pathway-gene network was highly clustered. The average degree (unweighted) of the network was 37.80. Among the 169 DEGs, 72 genes had a degree greater than 71. Only six genes were isolated, thus indicating that no other genes were involved in their specific pathways. The six genes were BnaA05g09600D, BnaA10g11530D, BnaA10g13870D, BnaAnng35070D, BnaC02g29620D and BnaC04g06610D. Two genes, BnaA08g31100D and BnaAnng27690D, displayed the most connections, connecting with the other 80 DEGs. In fact, the two DEGs with the largest number of connections were involved in pathways ko01110 (biosynthesis of secondary metabolites), ko00480 (glutathione metabolism), ko01100 (metabolic pathways), and ko00030 (pentose phosphate pathway), among which ko01110 and ko01100 were the two most prominent pathways. The degree distribution for the pathway-gene network is shown in Fig. 8(b), where the degrees roughly clustered into three groups. The three groups of nodes were inclusive of degrees from 0 to 10, 20 to 30, and 70 to 80. Nodes with degrees larger than 70 encompassed a large area. The pathway-gene network indicated that most of the DEGs had direct or indirect interactions through the same pathways. The clustering of the pathway-gene network indicated that the DEGs could be classified into different functional categories via preferred pathways.

The gene-pathway network (Fig. 8(c)) had an average degree (unweighted) of 4.52. There were nine isolated nodes in this network: ko00380 (tryptophan metabolism), ko00900 (terpenoid backbone biosynthesis), ko00909 (sesquiterpenoid and triterpenoid biosynthesis), ko03010 (ribosome), ko03050 (proteasome), ko03410 (base excision repair), ko04120 (ubiquitin-mediated proteolysis), ko04140 (regulation of autophagy), and ko04141 (protein processing in the endoplasmic reticulum), thus indicating that these pathways involved only one DEG. That is, these genes were involved in only one pathway. Pathways ko01110 (biosynthesis of secondary metabolites) and ko01100 (metabolic pathways) had the largest node degrees of 58 and 40, respectively, followed by ko00500 (starch and sucrose metabolism) and ko00940 (phenylpropanoid biosynthesis) with a node degree of 10. The distribution degrees for the gene-pathway network is shown in Fig. 8(d) (in log-log coordinates). The gene-pathway network was somewhat scale-free^45^, with a power-law exponent of approximately −1. The scale-free property of the network indicated that the gene-pathway network could withstand random attack, such that a random deletion in a pathway might not seriously damage the overall network. However, the network was susceptible to specific attack. For example, when the most connected pathways, ko01110 or ko01100, were eliminated from the network, the topology of the network would be severely damaged, thus indicating that the system had lost some of its functionality. Moreover, as shown in Fig. 8(a,c), the weights of some edges were far higher than others, thus indicating that some DEGs or pathways were concomitant with some other DEGs or pathways. For example, in Fig. 8(a), the weight between BnaC03g54190D and BnaC04g12030D was 6, which was the largest value obtained and indicated that the two DEGs shared 6 common pathways. In fact, the two DEGs all involved ko01110 (biosynthesis of secondary metabolites), ko01100 (metabolic pathways), ko00430 (taurine and hypotaurine metabolism), ko00250 (alanine, aspartate and glutamate metabolism), ko00650 (butanoate metabolism) and ko00410 (beta-alanine metabolism). The DEGs involved in more common pathways had more similar functions in life processes. Figure 8(c) shows that the edge between pathways ko01110 and ko01100 had the highest weight, equivalent to 41, thus indicating that 41 DEGs were simultaneously involved in the two pathways. An interplay was observed between pathways ko00940 (phenylpropanoid biosynthesis) and ko01110 via 12 common DEGs. The ko01100 and ko00500 (starch and sucrose metabolism), as well as the ko01100 and ko00940 pathways, all interacted with one another via 11 common DEGs. These findings indicated that some pathways might simultaneously involve a large number of common DEGs, and these pathways are likely to cross-talk and have overlapping biological functions.

Among the 169 DEGs, 156 were annotated to a total of 1191 unique biological processes. Through a sonstructed tripartite graph for the 169 DEGs, we obtained the pathway-process network and the process-pathway network, as well as their degree distributions, as shown in Supplementary Information Fig. S1. The average degree of the pathway-process network was 823.20 (980426 edges), thus indicating that each biological process was related to most of the other processes via shared pathways. A large amount of nodes (917/1191) had a degree higher than 800. The tight connectedness of the pathway-process network revealed that different biological processes participated in cross-talk with one another through common pathways. Moreover, the pathway-process network revealed a certain modularity indicating that the biological processes could be classified into functional groups; processes in the same group interacted with one another through preferred pathways. The process-pathway network had 85 nodes. The average degree of the network was 64.94 (5520 edges). The degree distribution revealed that more than 60 nodes had a degree higher than 70. Ten of the 85 pathways were isolated, and therefore were not simultaneously related to the same biological processes. The 10 isolated nodes included ko00860: porphyrin and chlorophyll metabolism, ko03430: mismatch repair, ko03440: homologous recombination, ko03018: RNA degradation, ko00240: pyrimidine metabolism, ko03030: DNA replication, ko03420: nucleotide excision repair, ko00310: lysine degradation, ko00350: tyrosine metabolism, and ko00071: fatty acid metabolism. Most of the isolated pathways were not enriched in any of the three categories. The process-pathway network for the 156 DEGs also indicated that most of the pathways participated in cross-talk with one another through common pathways, whereas several reduced pathways shared no common biological processes.

The process-gene network and gene-process network is shown in Supplementary Information Fig. S2. The gene-process network was tightly connected; there were more than 1.7 × 10^5^ edges among the 1191 nodes, with an average degree 295.13. The maximum degree was 1180, and the minimum degree was 14. Similarly, to the pathway-process network, the gene-process network also showed certain modularity, and it also indicated that the processes could be classified into different categories, which favored different DEGs. The degree distribution of the gene-process network revealed a scale-free property; a large number of nodes had a degree lower than 400, whereas a few nodes had a very large degree. The scale-free property of the network also indicated the gene-process network could withstand random mutations, which would not severely affect the biological function of the system. The process-gene network contained 13 isolated nodes and a densely connected component. Of the 169 nodes in the densely connected cluster, 152 had degrees higher than 100, whereas 27 DEGs exhibited the highest degree equal to 155. The isolated nodes currently lack biological process annotations. The edges between genes BnaA06g03560D and BnaC06g40170D displayed the highest weight of 158, and the weights between BnaA06g03560D and BnaA04g27940D and between BnaA06g03560D and BnaC06g40190D were all higher than 130, thus indicating their cross-talk with drought-responsive pathways by sharing many common biological processes.

### Quantitative real-time PCR validation

**Figure 9:**
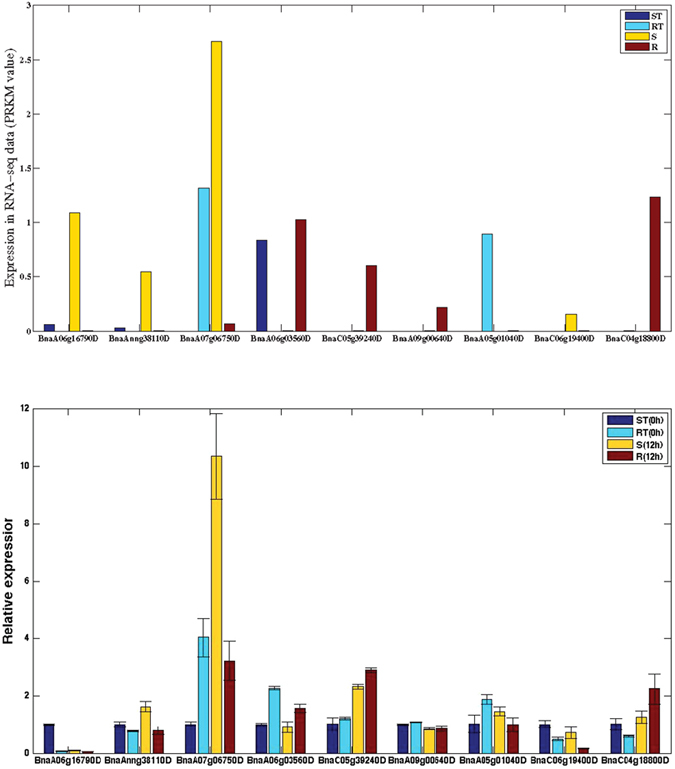
qRT-PCR validations on nine of the 169 crucial DEGs. The upper panel shows the expression levels of the nine genes in RNA-seq data (We only consider their expression in RT-R. S-R and ST-S). The lower panel shows the qRT-PCR results. The vertical axis shows the relative expression of the considered DEGs, the horizontal axis corresponds to the nine DEGs.

To confirm the accuracy and reproducibility of the Illumina RNA-Seq results, on the basis of the above analysis, nine representative genes were chosen, and their expression levels before and after the drought treatment were examined by quantitative real-time PCR (qRT-PCR) validation. See Materials and Methods for detailed experimental processes. A correlation between qRT-PCR and RNA-Seq was evaluated using fold-change measurements. The validation results for the nine genes are shown in Fig. 9.

Genes BnaC05g39240D, BnaA09g00640D and BnaC06g19400D were from CDR. BnaA07g06750D, BnaA05g01040D and BnaC04g18800D were from RDR. The final three genes (BnaA06g16790D, BnaAnng38110D and BnaA06g03560D) were from SDR. On the basis of the RNA-seq results, the three genes from CDR were all differentially expressed in S-R, in which BnaC05g39240D and BnaA09g00640D were up-regulated and BnaC06g19400D was down-regulated in R compared with S. BnaA07g06750D and BnaA05g01040D were down-regulated and BnaC04g18800D was up-regulated in R compared with RT. Moreover, BnaA07g06750D was also down-regulated in R compared with S. BnaA06g16790D and BnaAnng38110D were all down-regulated and BnaA06g03560D was up-regulated in R compared with S. The three genes from SDR were all expressed in ST-S. BnaA06g16790D and BnaAnng38110D were up-regulated and BnaA06g03560D was down-regulated in S compared with ST. As shown in Fig. 9, the relative trends in the expression patterns of the qRT-PCR results were all consistent with the RNA-Seq data, although there were some differences in the absolute expression levels.

## Discussion

Drought is a major adverse environmental factor that limits plant growth and agricultural productivity^12^. Drought stress and oxidative stress are often interconnected and may induce similar effects, such as cellular damage, and are thus difficult to control and engineer^11^,^12^. Stress-responsive genes can be used to improve stress tolerance in plants by gene transformation^7^,^8^,^34^,^46^. It is important to explore the functions of stress-responsive genes, not only to understand the molecular mechanisms responsible for stress tolerance and the responses of higher plants but also to improve the stress tolerance of crops through gene manipulation. Hundreds of genes are thought to be involved in abiotic stress responses^12^,^47^. Studies performed in recent decades have provided information about the exploration of drought resistance mechanisms in plants on morphological, physiological and molecular bases^7^,^9^.

We established two experimental conditions for two varieties of *B. napus* and performed transcriptomic analyses of four samples: R, RT, S, and ST. To screen drought-responsive DEGs, we constructed four comparison groups: RT-R, S-R, ST-RT and ST-S. The statistical analysis revealed 3545, 10346, 11055 and 1221 DEGs in RT-R, S-R, ST-RT and ST-S, respectively. By further excluding DEGs lacking known pathway annotations and selecting DEGs with large differences in expression levels among paired samples, 169 DEGs were finally selected as crucial drought-responsive DEGs. The current investigation has some limitations. On the one hand, during our selection of crucial DEGs, a large number of DEGs without known pathway annotations were not considered. The DEGs that currently lack pathway annotations might also be closely related to drought stress. Thus, one may use the idea of feature selection. For example, some biological processes are known to be closely related to drought stress, and thus DEGs without pathway annotations but involved in related biological processes can be selected for detailed investigations. On the other hand, during our selection of the 169 DEGs, we restricted the difference in expression levels above given thresholds. Thus, lowering the thresholds may provide more DEGs, although it remains difficult to determine how many DEGs are actually crucial drought-responsive ones. For example, excluding the selected 169 DEGs, BnaC07g44670D was differentially expressed between R and RT, with *log*2(*R*/*RT*) = 1.2528. In fact, BnaC07g44670D was homologous to gene ABF (AT4G34000) in *Arabidopsis thaliana*, which has been reported to be an important gene involved in ABA signaling. The up-regulation of ABF in response to drought stress can trigger stomatal closure and seed dormancy^7^, which is a typical response mechanism during drought stress.

In 2015, Zhang *et al*.^33^ reported 79 candidate genes involved in water tolerance in *B. napus*. Among these genes, only 34 were differentially expressed in the four samples in our experiment. Most of the 34 genes had very low absolute *log*2 fold change values in the four comparison groups in our experiment. Some of the genes were not identified with our selection criteria. Those genes either lacked a known pathway annotation or had high absolute *log*2(*RT*/*ST*) values. For example, for gene BnaC08g37930D, *log*2(*R*/*RT*) = −10.5097 and *log*2(*R*/*S*) = −11.8246, which are with very high absolute values. However, the absolute value of *log*2(*RT*/*ST*) was also higher than 1, and thus it is possible that this gene may be responsive to water treatment or responsible for clutivar differences. Although gene BnaC03g32950D demonstrated high absolute *log*2 fold changes, it lacked pathway annotations. Thus, these genes were not included as crucial drought-responsive DEGs. We analyzed potential explanations for the inconsistencies between our results and those reported by Zhang *et al*.^33^. First, the two studies utilized two different experiments. Zhang *et al*. was based on a GWAS study along with RNA-seq, without considering pathway annotations, whereas our study was based on RNA-seq of four samples. Another possible explanation is that many DEGs lacked known pathway annotations were therefore excluded from the subsequent analysis. It will be interesting to further verify the drought response of the identified genes in the two studies by using a new experimental design.

GO and KEGG pathway enrichment and involvement analysis were performed to reveal preferred GO terms or pathways for the selected DEGs. GO enrichment analysis revealed that the DEGs were enriched in response to various stimuli or stresses, such as abiotic, chemical, hormone, stress and water stimuli, osmotic stress, salt stress, other organisms, oxidative stress, starvation, water deprivation, and wounding. It is well-known that drought stress is a typical abiotic stimulus^26^, and enrichment for the DEGs in the abiotic stimulus illustrated that the selected DEGs might in fact be drought-responsive DEGs. The response to wounding may contribute to the repair of cell damage induced by water deficiency^21^. Osmotic stress responses may also be closely related to drought-responsive pathways. Osmotic adjustment is a crucial physiological mechanism for adaptation to water stress^48^, which helps maintain the dynamic balance between the damage and repair of cellular components to mitigate plant injury and enhance stress resistance abilities. Moreover, drought limits plant growth and development mainly through photosynthetic decline, osmotic stress-imposed constraints on plant processes, and interference with nutrient availability^27^. It has been reported that salt, drought, heat, cold stress and oxidative stress may be accompanied by the formation of ROS^49^, such as superoxide, hydrogen peroxide, and hydroxyl radicals, thus causing extensive cellular damage and inhibition of photosynthesis. This phenomenon is called oxidative stress and is one of the major causes of plant damage as a result of environmental stress^49^. Osmotic stress and the associated oxidative stress appear to be common consequences of exposure to drought and salinity^26^. ABA is a well-known plant hormone that functions in many plant developmental processes. ABA-mediated signaling plays an important role in plant responses to environmental stress and plant pathogens^50^,^51^. The response to the ABA stimulus is enriched in ST-S, S-R, and RT-R but reduced in ST-RT. As we have indicated, the DEGs in the comparison groups ST-S, S-R, and RT-R were all susceptible to drought stress, but ST-RT was not susceptible. Therefore, the enrichment for related processes in the selected DEGs further supports their drought responsiveness.

We further discussed several DEGs by analyzing typical related pathways. We explored plant hormone signal transduction and peroxisome pathways. The plant hormone signal transduction pathway is an important pathway. Plant hormones are organic substances that are synthesized in one tissue and transported to regions to provide a physiological responses. The main plant hormones include auxin, cytokinine, gibberellin, ABA, ethylene, brassinosteroid, and jasmonic acid. Hormone secretion can be stimulated and inhibited by environmental changes and other hormones, among other stimuli. Drought stress is a typical environmental stimulus that may induce plants to secrete hormones and activate plant hormone signal transduction pathways. Nine of the 169 DEGs were involved in plant hormone signal transduction pathways, including 3 RDR (BnaCnng59500D, BnaC03g12620D, BnaA07g06750D), 1 SDR (BnaA06g03560D) and 5 CDR (BnaC08g38290D, BnaA03g55400D, BnaA04g27940D, BnaC03g02620D, and BnaC01g41100D). Among the 9 DEGs, BnaA06g03560D and BnaCnn59500D were involved in the auxin signal transduction pathway, BnaC03g02620D and BnaA03g55400D were involved in the gibberellin signal transduction pathway, BnaA07go6750D was involved in the ethylene signal transduction pathway, BnaA04g27940D and BnaC08g38290D were involved in the brassinosteroid pathway, and the remaining two genes, BnaC01g41100D and BnaC03g12620D, participated in the jasmonic acid signal transduction pathway. The three DEGs in RDR were all down-regulated in R compared with RT, and BnaCnng59500D had the highest absolute R to RT fold change ratio (*log*_2_(*R*/*RT*) = −10.2858). BnaC03g12620D was also involved in plant circadian rhythms and plant-pathogen interactions. GO biological process annotations revealed that BnaCnng59500D, BnaA07g06750D and BnaC03g12620D were involved in 6, 43 and 45 processes, respectively. BnaCnng59500D and BnaA07g06750D participated in responses to various stimuli and compounds, whereas BnaC03g12620D participated in trichoblast differentiation, root epidermal cell differentiation, root morphogenesis, root development, root system development and many other developmental, regulatory and metabolic processes. The development of the root system can enhance drought tolerance.

**Figure 10:**
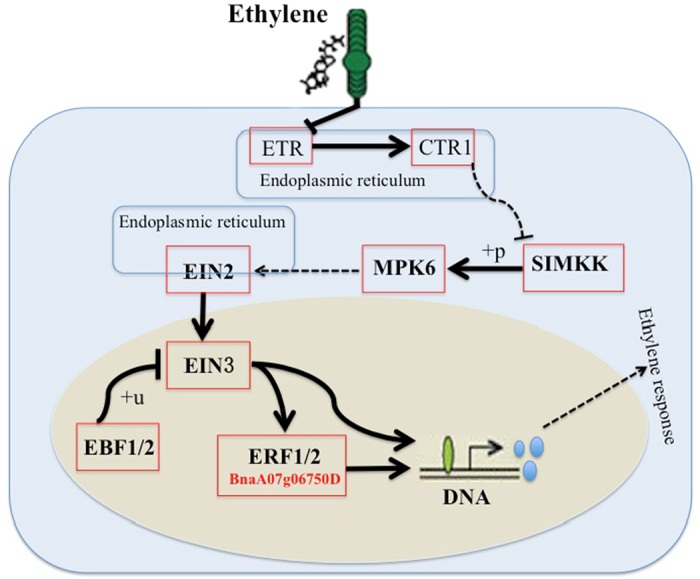
BnaA07g06750D involves in the plant hormone (Ethylene) signal transduction pathway in KEGG.

Ethylene is a well-known plant growth regulator that participates in a variety of stress and developmental processes^52^. Ethylene signaling components are likely to be conserved in responses to cell elongation, various stresses, cell fate patterning in the root epidermis, and fruit ripening^52^. BnaA07g06750D is homologous to the AT3G23230 gene in *Arabidopsis thaliana*, which is involved in the ethylene-response signal transduction pathway. BnaA07g06750D encodes a member of the ERF (ethylene response factor) subfamily: B-3 of the ERF/AP2 transcription factor family. The protein contains one AP2 domain. There are 18 members in this subfamily, including ERF-1, ERF-2, and ERF-5. ERF/AP2 has been shown to regulate developmental processes and the response of plants to various types of biotic and environmental stress^53^. In the ethylene-response signal transduction pathway, as shown in Fig. 10, EIN3 activates ERF1/2, and the products of EIN3 and ERF1/2 all function as transcription factors^54^, thus further activating the expression of genes and evoking an ethylene response, including the responses to cell elongation, various stresses, fruit ripening and senescence. The EIN3, ERF1/2 and downstream ethylene-responsive genes comprise a coherent feed-forward loop, which is a typical network motif and acts as a building block in complex biological systems^55^,^56^. The theoretical and experimental results showed that the coherent feed-forward loop reduces transduction noise and acts as a sign-sensitive delay in signal transduction^56^. ERF1/2 functions as a buffer, and drought signaling delays of the signaling system may represent another mechanism to cope with drought stress. Moreover, BnaA07g06750D is involved in 43 biological processes, including responses to various stimuli, compounds, and regulatory processes. Suzuki *et al*.^57^ have suggested that the expression of multiprotein bridging factor 1c (MBF1c) in Arabidopsis enhances the tolerance of transgenic plants to environmental stress by partially activating, or perturbing, the ethylene-response signal transduction pathway, and AT3G23230 has been identified as an important loci. In another study by Zhang *et al*.^58^, AT3G23230 has been found to enhance tolerance to salt through transcriptional activation of ascorbic acid synthesis in *Arabidopsis*. In our investigation, BnaA07g06750D was also identified as a very important drought-responsive gene, in agreement with the available information for its homologous *Arabidopsis thaliana* gene^57^,^58^.

Peroxisomes are organelles that are found in virtually all eukaryotic cells. They participate in the catabolism of very long chain fatty acids, branched chain fatty acids, D-amino acids, and polyamines, and ROS reduction. A major function of the peroxisome is the breakdown of very long chain fatty acids through beta-oxidation. The long fatty acids are converted to medium chain fatty acids, which are subsequently shuttled to mitochondria where they are eventually broken down into carbon dioxide and water. In plant cells, this process is carried out exclusively in peroxisomes^59^. Therefore, peroxisomes play an important role in metabolic processes. Two DEGs that are related to the peroxisome pathway were found in only CDR: BnaC05g39240D and BnaA09g00640D. These two DEGs were up-regulated in R compared with S. The *log*_2_ R to S ratios were all higher than 7.7. The two DEGs were involved in the biosynthesis of secondary metabolites and metabolic pathways. BnaC05g39240D was also involved in the glyoxylate and dicarboxylate metabolic pathways, and BnaA09g00640D was also involved in the purine metabolism and caffeine metabolism pathways. The two DEGs participated in 18 and 46 GO processes. BnaC05g39240D participated in responses to stress stimuli, biotic stimuli, single-organism metabolic processes, and defense responses, among others. The biological processes associated with BnaA09g00640D included responses to stimuli such as abiotic stimuli, floral organ morphogenesis, floral organ development, and flower development. As discussed in the Introduction, plants may cope with drought stress by a shortened life cycle with accelerated flowering, and the involvement of BnaA09g00640D in floral organ morphogenesis, floral organ development and flower development suggests that BnaA09g00640D may have some functional roles in drought responsiveness.

BnaC05g39240D is homologous to AT3G14420 (GOX1, peroxisomal (S)-2-hydroxy-acid oxidase GLO2) in *Arabidopsis thaliana*. Available information for AT3G14420 has revealed that the biogenesis of peroxisomes starts with the early peroxins PEX3, PEX16 and PEX19 and proceeds via several steps. The import of membrane proteins into peroxisomes requires PEX19 for recognition, targeting and insertion by docking at PEX3. Matrix proteins in the cytosol are recognized by peroxisomal targeting signals (PTS) and transported to the docking complex at the peroxisomal membrane. Peroxisome deficiencies lead to severe and often fatal inherited peroxisomal disorders. AT3G14420 encodes a glycolate oxidase that modulates ROS-mediated signal transduction during non-host resistance^60^. AT3G14420 is expressed during the seedling development stage, and it can affect plant growth and development. The available information for *Arabidopsis thaliana* provide evidence that AT3G14420 has functional roles during the stress response^60^,^61^,^62^. The homology between BnaC05g39240D and AT3G14420 indicates that BnaC05g39240D may have functional roles during the stress response. In summary, analysis of a few selected pathways indicated that associated pathways are putatively drought-responsive ones, and the related DEGs are actually involved in many biological processes that respond to drought stress.

Excluding the enrichment analysis of biological processes and pathways, we constructed six networks to reveal the relationships among different DEGs, pathways and biological processes. The pathway-gene network was found to be highly clustered, thus indicating that certain clusters of DEGs tended to participate in some similar pathways. BnaA07g06750D and BnaC05g39240D had 8 and 74 neighbors in the pathway-gene network, respectively. The degrees for these two genes were all very high. The plant hormone signal transduction and peroxisome pathways had 2 and 5 neighbors in the gene-pathway network. Plant hormone signal transduction was associated with plant-pathogen interactions with a weight of 3, thus indicating that the two pathways might participate in cross-talk. The gene-pathway network revealed that some pathways were connected with strong weights, and the degree distribution of the network follows power-law, which indicated that certain pathways preferentially functioned together and that the pathway network could withstand random mutations. The strong associations of the gene-process, the process-gene, the pathway-process and the process-pathway networks indicated that different processes or pathways had close relationships with one another. Notably, the constructed networks differed from the actual bio-molecular networks; the edges in the constructed networks indicated that the two nodes shared the same biological processes, pathways, or DEGs. Therefore, the constructed networks were able to reveal the functional relationships among different DEGs, processes and pathways.

## Conclusion

In this paper, we explored the transcriptomic basis for *B. napus* resistance to drought. On the basis of transcriptome sequencing of four experimental settings with drought-tolerant and drought-sensitive cultivars, statistical analysis of 6440 differentially expressed genes (DEGs) revealed three classes comprising a total of 169 crucial drought-responsive DEGs in *B. napus*. The three classes of DEGs consisted of 37 DEGs in RDR, 35 DEGs in SDR and 97 DEGs in CDR. The 169 drought-responsive genes were mostly well uniformly distributed throughout its 19 pairs of chromosomes, thus indicating that the chromosomal distribution of the crucial DEGs showed no location preference and that drought resistance might be a robust mechanism that evolved through natural selection. Most of the crucial DEGs were both down-regulated in the drought-resistant variety and up-regulated in the drought-sensitive variety after exposure to drought stress.

The three classes of crucial DEGs were frequently involved in certain processes and pathways, such as the response to various stimuli, metabolic processes, metabolic pathways, the biosynthesis of secondary metabolites, starch and sucrose metabolism, phenylpropanoid biosynthesis, and zeatin biosynthesis. The crucial DEGs in the drought-resistant variety also participated in plant hormone signal transduction, ABC transporters and oxidative phosphorylation pathways, whereas the flavonoid biosynthesis pathway showed specific involvement in the drought-sensitive variety. The GO and KEGG pathway analysis demonstrated that the crucial DEGs participated in some functional categories related to drought stress, and the associated pathways or processes had some functional roles in the response to drought stress.

qRT-PCR validation experiments supported the reproducibility of the associated results. Specific analysis of several homologous genes of *Arabidopsis thaliana* and related pathways further supported the importance of the selected DEGs for the response to drought stress. Clarification of crucial drought stress-responsive DEGs is fundamentally important for future transgenic research, and it also has important implications for the improvement of crop varieties.

## Materials and Methods

### Plant materials and RNA extraction

Two *B. napus* varieties with different types of resistance to drought stress were considered: 07Y19, which is resistant to drought (marked as RT), and 07Y29, which is sensitive to drought (marked as ST)^35^. The seeds were cultivated in 1/2 Hoagland medium with 16-h/8-h light/dark cycles. After germination for 7d, the seedlings were treated with 200 g *L*^−1^ PEG-6000 (marked as R and S), and RT and ST grown on 1/2 Hoagland medium served as controls. In the drought stress experiment, the rate of water loss was measured in leaves. Techniques to monitor drought stress are described in previous related works^35^,^36^,^37^,^38^,^63^. A previous study has shown that different genes that respond to drought stress are expressed within 0–48 h after drought treatment, and different genes are expressed at different times^35^. To induce drought stress-responsive gene expression, seedlings were sampled at 8 time points after treatment with PEG-6000 for RNA extraction: 0.5 h, 1 h, 3 h, 6 h, 9 h, 12 h, 24 h and 48 h. Each sample consisted of 10 individuals. Total RNA was extracted with TRIzol according to the manufacturer’s instructions (Invitrogen, USA). The concentration and quality of each RNA sample was determined using a NanoDrop 2000™ micro-volume spectrophotometer (Thermo Scientific, Waltham, MA, USA). Equal amounts of total RNA from different times and treatments of each sample were pooled to construct the cDNA library.

### Sequencing data

After total RNA extraction and DNase I treatment, magnetic beads with oligo(dT) were used to isolate mRNA. In a mixture with the fragmentation buffer, the mRNA was fragmented into 200–700-nt short fragments. The cDNA was then synthesized using mRNA fragments as templates. Short fragments were purified and resolved with EB buffer for end reparation and single nucleotide A (adenine) addition. Next, the short fragments were linked using adapters. Suitable fragments were selected as templates for PCR amplification.

Our primary sequencing data were produced using Illumina HiSeq™ 2000 and have been deposited in the NCBI Sequence Read Archive (SRA, http://www.ncbi.nlm.nih.gov/Traces/sra) under accession number SRP051461^35^. These data, denoted as raw reads, were subjected to quality control (QC) to determine whether a resequencing step was needed. Specifically, the raw reads were cleaned by removal of reads containing primer/adapter sequences, low-quality reads (i.e., a percentage of low quality bases greater than 50% in a read, with low quality bases defined as bases with a sequencing quality of no more than 10) and reads with more than 10% unknown bases. During the QC steps, an Agilent 2100 Bioanaylzer and ABI StepOnePlus Real-Time PCR System were used for the quantification and qualification of the sample library. The library was loaded onto the channels of an Illumina HiSeq™ 2000 instrument for 5 gigabase in-depth sequencing, which was used to obtain more detailed information about gene expression. Each paired-end library had an insert size of 200–700 bp. An average read length of 90 bp was generated as raw data.

After filtering, the remaining reads were called “clean reads”, which were aligned to the reference sequences with SOAPaligner/SOAP2^64^. No more than 5 mismatches were allowed in the alignment (SOAPaligner/SOAP2 recommends no more than 2 mismatches in a read length of 50 bp, and no more than 5 if the read length exceeds 90 bp). The alignment data were used to calculate the distribution of reads on reference genes, to perform coverage analysis and to proceed with the downstream analysis including gene expression, gene structure refinement, alternative splicing, novel transcript prediction and annotation and SNP detection.

### Gene expression level

The transcript abundance of each gene was estimated by using RPKM^42^ (reads per kilobase transcriptome per million mapped reads) with the following formula:


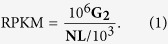


Because the expression of gene **G**_**1**_, **G**_**2**_ is the number of reads that are uniquely aligned to gene **G**_**1**_, **N** is the total number of reads that are uniquely aligned to all genes, and **L** is the number of bases in gene **G**_**1**_. The RPKM method is able to eliminate the influence of different gene lengths and sequencing discrepancies in calculations of gene expression levels. Therefore, the calculated gene expression level was able to be directly used to compare differences in gene expression among samples.

### Screening of differentially expressed genes

To identify genes with different expression levels among samples, on the basis of reference^65^, a strict algorithm to identify DEGs between two samples was developed as follows:

Denote the number of unambiguous clean tags (denoting RNA sequence reads) from gene **G**_**1**_ as *x*, suppose that the expression of each gene occupies only a small part of the library, and *x* yields the Poisson distribution:


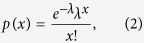


where *λ* denotes the real transcripts of the gene.

Assume the total clean tag number in sample 1 is **N**_**1**_, and the number in sample 2 is **N**_**2**_. Gene **G**_**1**_ holds *x* tags in sample 1 and *y* tags in sample 2. The probability of gene **G**_**1**_ being expressed equally between two samples can be calculated as follows:


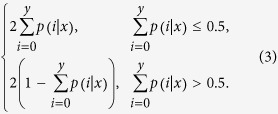


where


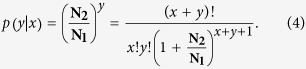


The P-value was obtained using the differential gene expression test. Because the DEG analysis generates numerous multiplicity problems in which thousands of hypotheses (whether gene *x* is differentially expressed between the two groups) can be tested simultaneously, corrections for false positive and false negative errors were performed using the false discovery rate (FDR) method^66^. Assume that we have selected *n* DEGs in which **m**_**1**_ genes show differential expression, and the other *m*_2_ genes are false positives. If we restrict the error ratio *F* = *m*_2_/*n* below a cutoff (e.g., 5%), we should preset the FDR to a number no larger than 0.05^66^. We used *FDR* ≤ 0.001^66^ and |*log*_2_(*Ratio*)| ≥ 1 as thresholds to determine the significance of gene expression differences. More stringent criteria with smaller FDR and higher fold-change values can be used to reduce the number of DEGs.

In our investigations, genes were coded as “BnaCXXgYYYYYD” or “BnaAZZgYYYYYD”^1^, in which “Bna” was the abbreviation for *B. napus* and “AZZ” or “CXX” represented the chromosome type and location (AZZ denotes chromosomes from *B. rapa*, and CXX from *B. oleracea*), where the label “XX” ranges from 01 to 09, “ZZ” ranges from 01 to 10, “Ann”, “Cnn” and “Unn” denote genes with unknown chromosome positions. A number with five characters, “YYYYY” denotes the location of a gene on the chromosome.

### Filtering crucial DEGs

We designed four comparison groups: RT versus R (RT-R), S versus R (S-R), ST versus RT (ST-RT), and ST versus S (ST-S), and we quantitatively determined the DEGs by transcriptome sequencing of each comparison group. The detailed experimental design can be found in our previous work^35^ or others therein^67^. The filtering processes were as follows. First, we omitted genes with *log*2 expression levels lower than 1 and *FDR* < 0.001. Then, on the basis of the constructed four comparison groups, ST-S, S-R, ST-RT, and RT-R, we compared the relative *log*2 fold change values in each group for each gene. Only those DEGs with large fold-change ratios (see Table 3) and low FDR (*FDR* < 0.001) were considered to be candidate genes. We noted that larger *log*2 fold change values indicated a stronger response to drought stress. For example, if *log*2(*R*/*RT*) was very large, then the gene expression levels in R and RT displayed a larger difference, thus indicating that the gene had a strong response to drought stress.

### GO and KEGG pathway enrichment analysis

The GO encompasses cellular components, molecular functions and biological processes. In this paper, we mainly considered biological process, and the enrichment analysis was performed with GO::TermFinder^43^, using the hypergeometric test for statistical analysis with the whole *B. napus* transcriptome as the background. For *P* value correction (to control the false discovery rate *FDR*), the rigorous Bonferroni correction method^68^ was used. We considered the corrected *P*-value *FDR* ≤ 0.05 as a threshold. GO terms fulfilling this condition were defined as significantly enriched terms. This analysis was able to recognize the main biological functions or processes associated with the concerned DEGs.

Genes usually interact with one another in certain biological functions. KEGG pathway enrichment analysis helps to further understand the biological functions of genes. This analysis identifies significantly enriched metabolic pathways or signal transduction pathways in DEGs in comparison to the whole genome background. KEGG^44^ is used to perform pathway enrichment analysis of DEGs. The method used for the calculations was similar to the GO enrichment analysis.

### Network construction

**Figure 11:**
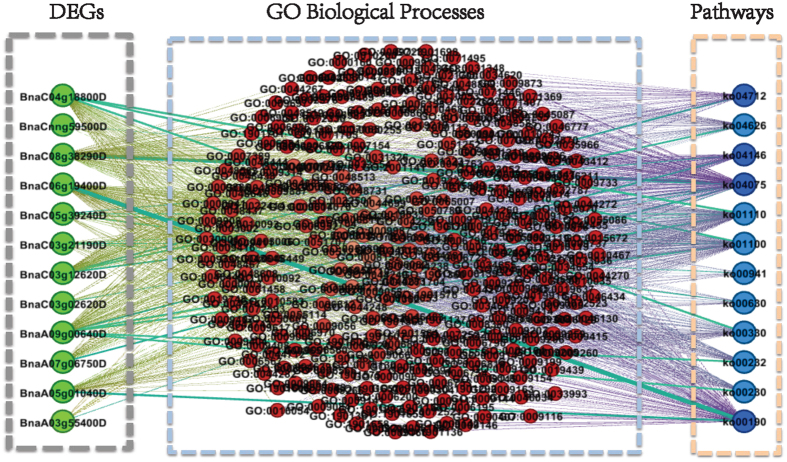
Graph for selected 12 DEGs, related GO biological processes and pathways. If a DEG relates to a biological process and a pathway, then connect the DEG and the biological process, the DEG and the pathway, as well as the biological process and the pathway.

To reveal the internal relationships among the selected DEGs and their biological processes and pathways, we constructed networks and performed network analysis^19^,^45^,^69^. First, we constructed a tripartite graph that mapped the DEGs to their biological processes and pathways. In detail, if a DEG was involved in some biological processes and pathways, then we connected the DEG with each of its biological process terms and pathway terms. Moreover, we connected each biological process term with each pathway term related to the same DEG. Thus, we were able to derive a tripartite graph containing three kinds of nodes: DEGs, biological process terms and pathway terms. For example, we selected 12 DEGs and generated the related tripartite graph, as shown in Fig. 11.

The 12 DEGs played roles in 313 unique biological processes and 12 unique pathways. The three kinds of nodes in the tripartite graph corresponded to 12 DEGs, 313 biological process terms and 12 pathway terms. If two DEGs were simultaneously involved in the same pathway, then we connected the two DEGs with an edge and thus derived a weighted pathway-gene network with nodes representing DEGs, where the weight of an edge denoted the number of pathways common to the two DEGs. Similarly, if two DEGs were simultaneously involved in the same biological process, we also connected them and ultimately obtained a process-gene network. Notably, although the nodes in the pathway-gene network and process-gene network all represented DEGs, they had different topological structures. The edges of the two networks had different biological meanings. Similarly, we constructed a gene-pathway network, a process-pathway network, a pathway-process network and a gene-process network. The six networks from the tripartite graph were used to reveal relationships among DEGs, biological processes and pathways.

Following the example of the 12 DEGs, on the basis of the constructed tripartite graph for the 169 DEGs, we were able to construct a gene-pathway network and pathway-gene network, gene-process network and process-gene network, and a process-pathway network and pathway-process network. The constructed networks are presented in Fig. 8 and Supplemental Figs S1 and S2.

### qRT-PCR

The qRT-PCR was conducted using GoTaq qPCR Master Mix (Promega Biotechnology) on an ABI 7500 FAST real-time PCR machine (Applied Biosystems, USA) with a final volume of 10 *μL* per reaction. Each reaction mixture contained 5 *μL* GoTaq Mix (GoTaq qPCR Master Mix 2X), 2.0 *μL* cDNA template, 0.5 *μL* each primer (1.0 *μM*), and 2 *μL* nuclease-free water. Each reaction was performed in triplicate. The cycling parameters were 95 °C for 5 min, followed by 40 cycles at 95 °C for 15 s and 60 °C for 30 s. Melt-curve analyses were performed using a program of 95 °C for 15 s and then a constant increase from 60 °C to 95 °C. The *B. napus* Actin1 gene was used as the internal reference gene. The relative gene expression levels were determined using the 2^−Δ*Ct*^ method^70^.

## Additional Information

**How to cite this article**: Wang, P. *et al*. Transcriptomic basis for drought-resistance in Brassica napus L. *Sci. Rep.* **7**, 40532; doi: 10.1038/srep40532 (2017).

**Publisher's note:** Springer Nature remains neutral with regard to jurisdictional claims in published maps and institutional affiliations.

## Supplementary Material

Supplementary Information

Supplementary S1_Table

Supplementary S2_Table

Supplementary S3_Table

Supplementary S4_Table

## Figures and Tables

**Table 1:** Statistics of transcriptome sequencing results for the four samples.

Sample	R	RT	S	ST
Total clean reads	66710312	68854546	67752586	68530142
Genome map rate	78.53%	78.29%	77.39%	77.42%
Gene map rate	61.77%	60.73%	60.04%	62.00%
Expressed gene	66709	68005	66149	66368
Novel transcripts	3567	3709	3316	3222
Alternative splicing	61554	68039	64040	62811
Gene fusion	−1	−1	−1	−1

**Table 2:** Classification of the possibly drought stress responsive DEGs.

Categories	Subgroups	Number of DEGs	Descriptions
RDR	Only RT-R;	487	Related to resistant cultivar
	RT-R, S-R	282	
SDR	Only ST-S;	111	Related to sensitive cultivar
	S-R, ST-S	114	
CDR	Only S-R;	1086	Cultivar nonspecific
	RT-R, ST-S;	85	
	RT-R, S-R, ST-S	20	

**Table 3:** Lists of the 169 crucial drought responsive DEGs.

Category	Subgroup	Conditions	N	Labels of crucial DEGs
RDR	RT-R	|*log*_2_(*R*/*RT*)| ≥ 5	21	BnaC03g56210D, BnaCnng59500D, BnaA05g01040D, BnaCnng20790D, BnaC09g44020D, BnaC03g12620D, BnaC01g37110D, BnaA02g18900D, BnaC07g19090D, BnaA05g28740D, BnaC05g09790D, BnaA10g07930D, BnaC01g34960D, BnaA06g03410D, BnaA03g24880D, BnaA03g03260D, BnaA02g11330D, BnaA03g34560D, BnaC03g58100D, BnaC09g15390D, BnaC04g18800D
RDR	RT-R, S-R	|*log*_2_(*R*/*RT*) − *log*_2_(*R*/*S*)| ≥ 2 or |*log*_2_(*R*/*RT*)| ≥ 5 or |*log*_2_(*R*/*S*)| ≥ 5	16	BnaA03g58830D, BnaC04g08480D, BnaAnng21300D, BnaA08g28220D, BnaC01g17200D, BnaA03g06740D, BnaA08g14940D, BnaC01g15100D, BnaA07g06750D, BnaA10g13870D, BnaC04g25850D, BnaAnng32880D, BnaA06g30800D, BnaC02g09490D, BnaC04g24050D, BnaA03g51150D
SDR	ST-S	|*log*_2_(*S*/*ST*)| ≥ 5	3	BnaA09g29930D, BnaA01g15420D, BnaA10g19500D
SDR	S-R, ST-S	|*log*_2_(*R*/*S*) − *log*_2_(*S*/*ST*)| ≥ 5 or |*log*_2_(*R*/*S*)| ≥ 5 or |*log*_2_(*S*/*ST*)| ≥ 5	32	BnaC04g53040D, BnaA07g25920D, BnaC03g21190D, BnaA06g16790D, BnaAnng38110D, BnaC07g23420D, BnaA05g07610D, BnaC09g30400D, BnaA05g04050D, BnaC01g15930D, BnaA10g25810D, BnaA08g10720D, BnaC08g34950D, BnaAnng13920D, BnaA05g01210D, BnaC06g27540D, BnaA09g45070D, BnaA02g35770D, BnaCnng49200D, BnaAnng16550D, BnaC01g01680D, BnaC01g00790D, BnaC04g12030D, BnaC07g22910D, BnaC09g13640D, BnaC04g40560D, BnaAnng35070D, BnaC06g40190D, BnaA06g03560D, BnaA08g15660D, BnaCnng58050D, BnaA08g15380D
CDR	S-R	|*log*_2_(*R*/*S*)| ≥ 5	72	BnaC04g08380D, BnaC08g34220D, BnaC09g25150D, BnaC04g06610D, BnaA05g36130D, BnaA07g09990D, BnaC06g40170D, BnaC03g54190D, BnaA04g19170D, BnaC06g42330D, BnaC01g32000D, BnaA03g18320D, BnaA03g17650D, BnaA06g25710D, BnaCnng24430D, BnaC03g65980D, BnaA10g01810D, BnaA10g23290D, BnaC02g29620D, BnaC04g04810D, BnaA05g25890D, BnaA02g15580D, BnaC06g19400D, BnaA09g13870D, BnaA09g13340D, BnaA01g27050D, BnaC03g71340D, BnaA06g20540D, BnaC04g47740D, BnaA09g00640D, BnaA07g30770D, BnaC02g05370D, BnaC07g47180D, BnaC06g27550D, BnaAnng19400D, BnaA03g23600D, BnaA02g05950D, BnaC08g38290D, BnaCnng16640D, BnaC01g41100D, BnaC02g43320D, BnaA01g11120D, BnaC09g40740D, BnaC04g07040D, BnaC04g47180D, BnaC05g39240D, BnaC08g07260D, BnaC04g51180D, BnaC03g10010D, BnaA06g17920D, BnaC03g43240D, BnaC03g28890D, BnaCnng65600D, BnaC09g42520D, BnaC04g19980D, BnaAnng28200D, BnaC01g25210D, BnaC09g54060D, BnaA02g30890D, BnaC04g30340D, BnaA04g09660D, BnaC06g20800D, BnaC03g15560D, BnaA05g04030D, BnaA07g09660D, BnaA04g11170D, BnaA02g32580D, BnaC04g48100D, BnaC03g30200D, BnaAnng33710D, BnaC05g20880D, BnaC09g30440D
CDR	RT-R, ST-S	|*log*_2_(*R*/*RT*) − *log*_2_(*S*/*ST*)| ≥ 1 or |*log*_2_(*R*/*RT*)| ≥ 3 or |*log*_2_(*S*/*ST*)| ≥ 3	14	BnaA05g24610D, BnaA03g36420D, BnaA04g15830D, BnaAnng27690D, BnaA05g09600D, BnaA03g55400D, BnaC05g04100D, BnaA02g16190D, BnaA08g31100D, BnaC09g21910D, BnaA03g46430D, BnaC09g28340D, BnaC03g42310D, BnaA09g55800D
CDR	RT-R, S-R, ST-S	|*log*_2_(*R*/*RT*)| ≥ 2 or |*log*_2_(*R*/*S*)| ≥ 2 or |*log*_2_(*S*/*ST*)| ≥ 2	11	BnaA04g17930D, BnaA04g27940D, BnaA05g27700D, BnaA08g04230D, BnaA09g41620D, BnaA10g11530D, BnaC01g39170D, BnaC02g00830D, BnaC03g02620D, BnaC04g22390D, BnaC09g48690D,

N: Number of DEGs.
