# Supplementary material for: Transcriptomic basis for drought-resistance in Brassica napus L
Source: Sci Rep. 2017 Jan 16;7:40532. doi: 10.1038/srep40532 (PMC5238399; doi:10.1038/srep40532)
Supplement: Supplementary Information [file srep40532-s1.pdf]

# Supplementary information for:

# Transcriptomic basis for drought-resistance in Brassica napus L.

Pei Wang, Cuiling Yang, Hao Chen, Chunpeng Song, Xiao Zhang, and Daojie Wang*

State Key Laboratory of Cotton Biology; School of Mathematics and Statistics; Henan Key Laboratory of Plant Stress Biology; College of Life Sciences; Henan University, Kaifeng, Henan, China

*Correspondence and requests for materials should be addressed to D. Wang (wangdj@henu.edu.cn)

## Supplementary datasets

**1. Supporting file 1: Tab S1.xls.**

DEGs in the four comparison groups with known pathway annotations. After filtering out the DEGs without known pathway annotations, only 1491, 4074, 4255 and 515 DEGs are left in RT-R, S-R, ST-RT and ST-S, respectively.

**2. Supporting file 2: Tab S2.xls.**

The detailed information of the selected 169 DEGs. The 169 DEGs are classified into three categories. 37 DEGs are RDR, 35 DEGs are SDR and 97 DEGs are CDR. Detailed information, such as $log_2(ratio)$, GO terms and pathways for each DEGs are given in this file.

**3. Supporting file 3: Tab S3.xls.**

KEGG pathway enrichment analysis for the 3545 DEGs in RT-R, 10346 DEGs in S-R, 11055 DEGs in ST-RT and 1221 DEGs in ST-S.

**4. Supporting file 4: Tab S4.xls**

KEGG pathway enrichment analysis for the selected 37 DEGs in RDR, 35 DEGs in SDR and 97 DEGs in CDR.

## Supplementary figures

### 5. Supporting file 5: Fig S1

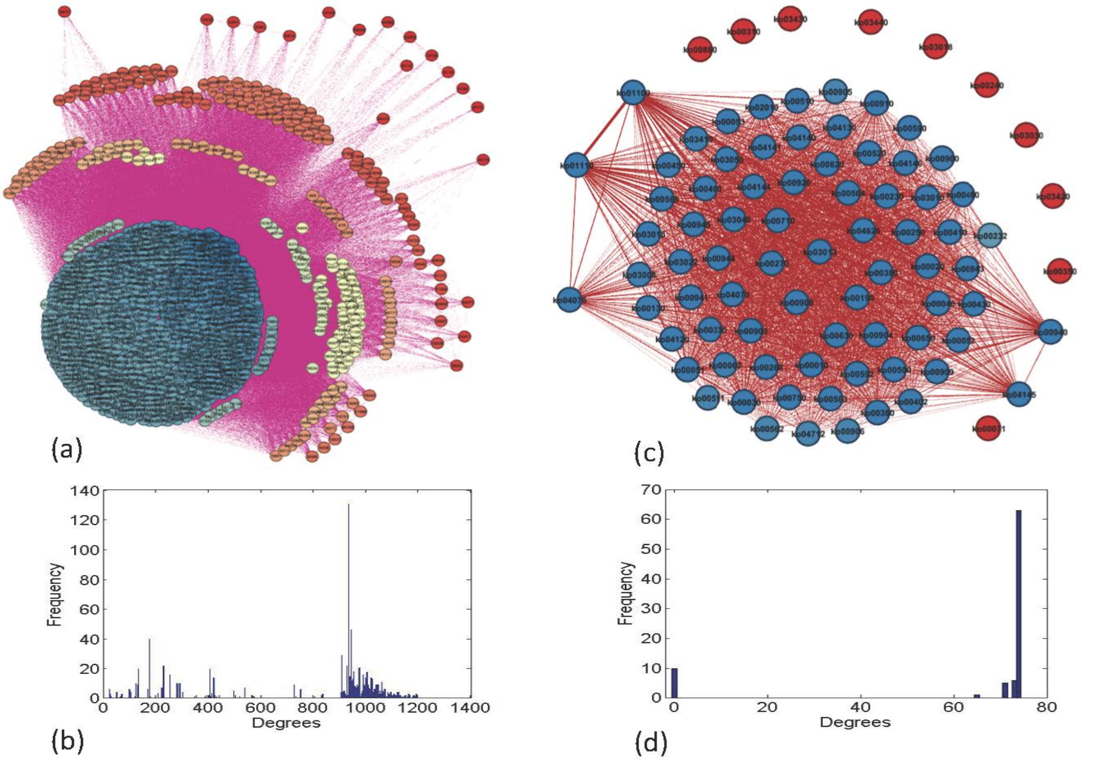


Fig.S1.The pathway-process network and the process-pathway network for 156 of the169 crucial biological process annotated DEGs. (a)The pathway-process network. Nodes correspond to the 1191 unique processes, if two processes relate to one or more pathways for the same DEGs, we endow a connection between them. Nodes with different colors are with different degrees. (b) Degree distribution for the pathway-process network in (a). (c) The process-pathway network for the 85 unique pathways that the 156 crucial biological process annotated DEGs participated. Nodes represent the 85 pathways, if two pathways involved in the same biological process for the same DEGs, then connect the two pathways by an edge. Numbers in panel (c) represent the pathway numbers. The thickness of an edge between two node in the two networks is proportion to the number of common DEGs involve in the two pathways. (d) The corresponding degree distribution of the process-pathway network as shown in (c).

**6. Supporting file 6: Fig S2**

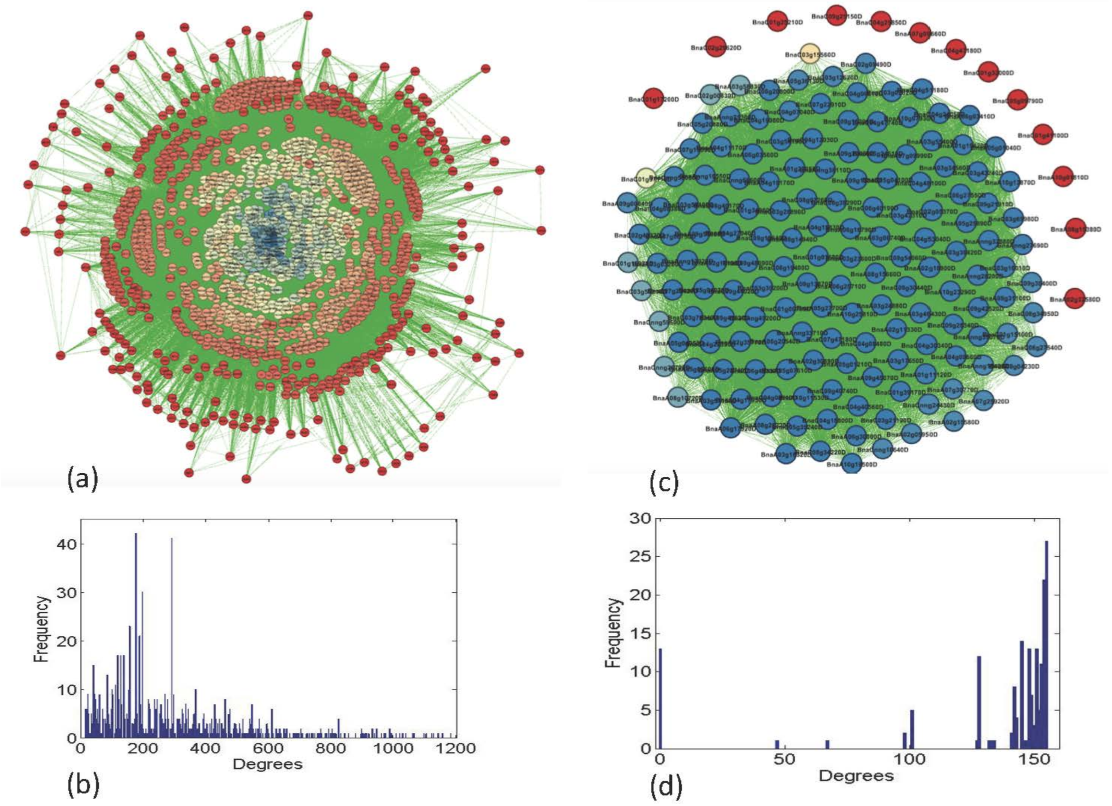


Fig. S2. The gene-process network and the process-gene network. (a)The gene-process network, nodes correspond to the 1191 unique processes, edges represent there are common DEGs involve in the two processes. (b) Degree distribution of the network as shown in (a). (c)The process-gene network. Nodes correspond to the 169 unique genes, if two genes share one or more process for the same DEGs, we endow a connection between them. Nodes with different colors are with different degrees. (d) Degree distribution for the process-gene network in (c).
